# Discrimination of the Essential Oils Obtained from Four Apiaceae Species Using Multivariate Analysis Based on the Chemical Compositions and Their Biological Activity

**DOI:** 10.3390/plants10081529

**Published:** 2021-07-26

**Authors:** Dilafruz N. Jamalova, Haidy A. Gad, Davlat K. Akramov, Komiljon S. Tojibaev, Nawal M. Al Musayeib, Mohamed L. Ashour, Nilufar Z. Mamadalieva

**Affiliations:** 1 Institute of Botany of the Academy Sciences of Uzbekistan, Durmon Yuli Str. 32, Tashkent 100125, Uzbekistan; dilafruz.jamalova.91@mail.ru (D.N.J.); ktojibaev@mail.ru (K.S.T.); 2 Department of Pharmacognosy, Faculty of Pharmacy, Ain Shams University, Cairo 11566, Egypt; haidygad@pharma.asu.edu.eg; 3 Department of Pharmacognosy, Faculty of Pharmacy, King Salman International University, South Sinai 46612, Egypt; 4 Institute of the Chemistry of Plant Substances, Academy of Sciences of RUz, Mirzo Ulugbek Str. 77, Tashkent 100170, Uzbekistan; a.davlat@inbox.ru; 5 Department of Pharmacognosy, College of Pharmacy, King Saud University, Riyadh 11495, Saudi Arabia; nalmusayeib@ksu.edu.sa

**Keywords:** Apiaceae, *Elaeosticta allioides*, *Elaeosticta polycarpa*, *Ferula clematidifolia*, *Hyalolaena intermedia*, essential oils, biological activity

## Abstract

The chemical composition of the essential oils obtained from the aerial parts of four Apiaceae species, namely *Elaeosticta allioides* (EA), *E. polycarpa* (EP), *Ferula clematidifolia* (FC), and *Hyalolaena intermedia* (HI), were determined using gas chromatography. Altogether, 100 volatile metabolites representing 78.97, 81.03, 85.78, and 84.49% of the total components present in EA, EP, FC, and HI oils, respectively, were reported. allo-Ocimene (14.55%) was the major component in FC, followed by D-limonene (9.42%). However, in EA, germacrene D (16.09%) was present in a high amount, while heptanal (36.89%) was the predominant compound in HI. The gas chromatographic data were subjected to principal component analysis (PCA) to explore the correlations between these species. Fortunately, the PCA score plot could differentiate between the species and correlate *Ferula* to *Elaeosticta* species. Additionally, the antioxidant activity was evaluated in vitro using the 2,2-diphenyl-1-picryl-hydrazyl-hydrate (DPPH), 2,2-azino-bis(3-ethylbenzothiazoline-6-sulphonic acid) (ABTS), and the ferric reducing power (FRAP) assays. In addition, the antimicrobial activity using the agar diffusion method was assessed, and the minimum inhibitory concentrations (MICs) were determined. Furthermore, the cell viability MTT assay was performed to evaluate the cytotoxicity of the essential oils against hepatic (HepG-2) and cervical (HeLa) cancer cell lines. In the DPPH assay, FC exhibited the maximum activity against all the antioxidant assays with IC50 values of 19.8 and 23.0 μg/mL for the DPPH and ABTS assays, respectively. *Ferula* showed superior antimicrobial and cytotoxic activities as well. Finally, a partial least square regression model was constructed to predict the antioxidant capacity by utilizing the metabolite profiling data. The model showed excellent predictive ability by applying the ABTS assay.

## 1. Introduction

Central Asia, particularly its hilly regions, is one of the richest and significantly diverse areas for the growth of family Apiaceae in the world. Kazakhstan, Kyrgyzstan, Tajikistan, Turkmenistan, and Uzbekistan are the habitats for about 458 species belonging to 110 genera of this family [1]. Interestingly, the Uzbek flora includes 231 species belonging to the family Apiaceae [2,3]. Many well-known plants belong to this family are widely used in culinary and therapeutic purposes such as anise, asafoetida, caraway, carrot, celery, coriander, cumin, dill, fennel, parsley, and many others. These plants are distinguished by the presence of volatile components that have long been regarded as scents with healing powers for body, mind, and soul with minimum side effects. Most of these essential oils’ components are potent antibacterial, antiviral, and antifungal agents [4].

Uzbekistan has approximately 100 species of *Elaeostica*, *Ferula*, and *Hyalolaena*, with nearly 25% of them being endemic and not found in any other places [5]. The local healers use many of these plants to treat infectious conditions, and they can also be used for culinary purposes [6]. However, the paucity of scientific data on their uses and concern that most of these plants may be endangered due to their massive consumption [7] initiated our interest in documenting their chemical compositions and biological activities.

The genus *Hyalolaena* Bunge is distributed around the lower Syr Darya river. Mountainous Central Asia is the focal center of diversity for *Hyalolaena*, of which nine species are widely distributed. Three species, *H. bupleuroides* (Schrenk) Pimenov & Kljuykov, *H. jaxartica*, and *H. trichophylla* (Schrenk) Pimenov & Kljuykov, have broad distribution ranges. Two species of *Hyalolaena*, namely *H. bupleuroides* and *H. intermedia*, are newly recorded in Uzbekistan [8]. These species are perennial monocarpic herbs with tuberiform, globose, obovate, cylindrical, or cordlike roots. *Hyalolaena intermedia* Pimenov and Kljuykov grows on rocks, red clay outcrops, clay-gravelly slopes, granite rocks at an altitude of 800–1200 m above sea level. It is distributed in the Western Tien Shan region, located in Kyrgyzstan and Uzbekistan [2].

*Elaeosticta* Fenzl is native to Ukraine to Central Asia and Western Himalayas. This genus contains at least 26 species [2,9], of which 15 species grow in Uzbekistan. *Elaeosticta allioides* (Regel & Schmalh.) Kliuikov, Pimenov, and V. N. Tikhomirov is a perennial, monocarpic plant common in Central Asia and Northern Iran. It grows on soft slopes, less often on rocky terraces and channels, in belts of semi-deserts, and on dry grassy steppes at an altitude of 500–2100 m above sea level sedge-bluegrass. It blooms in May-June and bears fruits in July-August. *Elaeosticta polycarpa* (Korovin) Kliuikov, Pimenov, and V. N. Tikhomirov is common in Central Asia and Afghanistan. It grows in the foothills on soft grassy slopes in the belt of the ephemeral semi-desert on the border (Angren region). It blooms in May, bears fruits in June, and is mainly used as fodder. Many studies revealed that the aerial part of this species, which showed antibacterial activity, contains essential oil, while in the roots, saponins are common [10].

*Ferula* L. includes about 200 species of flowering plants. Many of these species are utilized for medicinal, nutritional, and fodder purposes. Most species of this genus contain a high number of essential oils and resins. There are 114 species in Central Asia and about 60 in Uzbekistan, of which 5 are endemic [11,12]. These species grow mainly in the mountains at relatively high altitudes at a level of 300 to 3600 m above sea level, both on fine-grained, variegated layers and gravelly slopes. These plants contain essential oils or resin-like substances, coumarins, flavonoids, and (rarely) saponins in their organs [12,13]. In the same context, *F. clematidifolia* Koso-Pol is a perennial, polycarpic plant distributed in the Pamir-Alai (Zeravshan ridge, Takhta-karacha, Hissar, Karategin, Babatag). It blooms in May-June and bears fruits in June-August. Some of these endemic species of *Ferula* were evaluated for their antioxidant and anti-inflammatory activities. The results showed that most *Ferula* species had moderate antioxidant activity [14].

There are no studies on the volatile components and the essential oils’ compositions of *Elaeosticta allioides*, *E. polycarpa*, *Ferula clematidifolia*, and *Hyalolaena intermedia* growing in Uzbekistan. This study investigates the chemical composition of these species based on gas chromatography/mass spectrometry (GC-MS) analyses. Besides, chemotaxonomic discrimination between these species was conducted by applying multivariate analysis. In addition, the essential oils were evaluated for their antioxidant, antibacterial, and cytotoxic activities. Finally, a partial least square (PLS) regression model was constructed to investigate the correlation between the chromatographic fingerprint and the antioxidant activity.

## 2. Results and Discussion

### 2.1. Chemical Composition Determination

The chemical compositions of the essential oils collected from the aerial branches of four Apiaceae plants were determined by qualitative and semi-quantitative analyses using gas chromatography (GC) techniques. All of the oils are typical yellow with distinct smells. One hundred metabolites were identified (Table 1), representing 78.97, 81.03, 85.78, and 84.9% of the detected components in *E. allioides*, *E. polycarpa*, *F. clemaditifolia*, and *H. intermedia* essential oils, respectively.

These essential oils demonstrated a high degree of metabolic variability, both qualitatively and quantitatively. allo-Ocimene was the main compound in *F. clemaditifolia*, with the highest percentage (14.55%), followed by D-limonene (9.42%) and tetrahydro-linalool (9.02%). In *E. allioides*, germacrene D (16.09%), caryophyllene acetate (4.25%), α-bisabolol (4.17%), and β-bisabolenal (4.07%) were representing the major identified components. However, 3-methylenecyclohexene (9.46%), hinokiol (7.72%), and p-mentha-1,4-dien-7-ol (6.71%) were the main compounds detected in *E. polycarpa*. Concerning, *H. intermedia*, heptanal (36.89%), (3Z)-octen-1-ol (4.73%), and (E)-2-nonenal (4.24%) were the predominant compounds.

Monoterpenes and oxygenated monoterpenes are the predominant classes of metabolites detected in the *F. clemaditifolia* essential oil, where they represent 42.28 and 31.05% of the total components, respectively. Similar results could be noticed with these classes in many *Ferula* species. For instance, terpinolene (77.62%) was the major component in *F. macrecolea* [15], while β-pinene (60.84%) was the predominant metabolite in *F. gummosa* [16]. In *F. tunetana*, α-pinene (39.8%) was the main component [17] and α-thujene (13.5%) is present in the highest concentration in *F. tingitana* [18]. On the same context, these results were following our previous studies on other *Ferula* species where D-limonene was a major identified component in 6 different *Ferula* species [19]. These diverse metabolic profiles of *Ferula*, can be attributed to the wide spread of the genus in many continents and the subsequent great variability in the climatic and the soil characteristics, which have a great impact on the essential oils composition in the plants [20].

Oxygenated monoterpenes are also the main class in *E. polycarpa*, representing 19.7% of the detected components in its essential oil. However, oxygenated sesquiterpenes are the dominant class of metabolites in *E. allioides* (34.45%). Rare studies were carried out on this small genus. Hamedi et al. studied the essential oil composition of *E. glaucescens* [21]. The aliphatic ester constituted 52.0%, followed by the sesquiterpene hydrocarbon (6.1%), which are the major reported classes of the secondary metabolites. Conversely, aldehyde and ketones are highly predominant in *H. intermedia*, accounting for 53.79% of the identified components. It was the first report on the chemical composition of the volatile components in the genus.

### 2.2. Multivariate Data Analysis

#### Explorative Data Analysis

A useful approach for differentiation between closely related species is applying multivariate analysis based on various chromatographic techniques [22,23]. Multivariate analysis was used based on the GC data to determine the relationships between these Apiaceae species and explore similarities and dissimilarities between them. Principal Component Analysis (PCA) was primarily used to categorize data and to find correlations between samples and variables. After that, Hierarchical Cluster Analysis (HCA) was used for sorting samples into clusters based on their vicinity to each other. The relative peak areas of all compounds in the chromatograms from the studied species were subjected to both PCA and HCA analyses.

**Figure 1:**
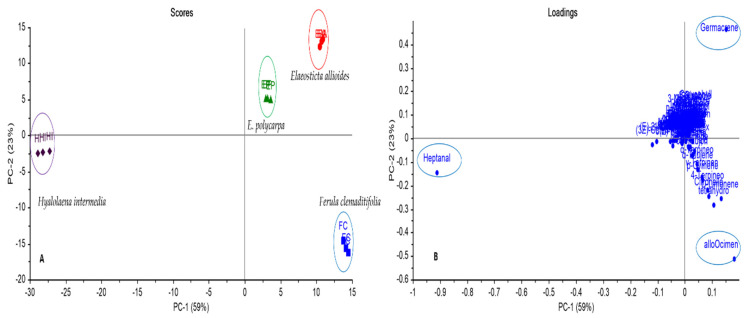
Principal Component Analysis score plot (**A**) and loading plot (**B**) of GC-MS analysis of essential oils of different Apiaceae species based on the identification of compounds shown in Table 1. *E. allioides* (EA), *E. polycarpa* (EP), *F. clemaditifolia* (FC), and *H. intermedia* (HI).

The score and loading plots for the data set are shown in Figure 1. The PCA scores plot for principal components (PCs), namely PC1 versus PC2, is shown in Figure 1a. The total variance of 82% was explained with two PCs. Different Apiaceae species could be discriminated from each other into three main groups, where they are positioned at three different quadrants. Both *F. clemaditifolia* and *H. intermedia* were positioned in separate quadrants highlighting their obvious dissimilarity from the remaining species. However, the PCA score plot showed *F. clemaditifolia* closeness to genus *Elaeosticta*, as both were positively correlated to PC1. This correlation may be attributed to the chemotaxonomic similarity due to common components such as D-limonene, dihydro-linalool acetate, hexadecanoic acid, phytol, methyl stearate, and ethyl octadecanoate. This correlation is quite interesting as it is the first chemotaxonomic classification carried out on these two species. Most morphological and anatomical classifications typically correlate both *Hyalolaena* and *Elaeosticta* genera together, and *Ferula* is usually separated [24]. These results encourage researchers to perform more studies on the chemotaxonomy of Apiaceae. Moreover, *E. allioides* and *E. polycarpa* were falling together in the same upper quadrant distanced from each other (as both are from the same genus). By clear inspection of the loading plot Figure 1b, it revealed the effective variables for each PCs where allo-ocimene, heptanal, and germacrene D were the key makers accountable for the segregation of *F. clemaditifolia*, *H. intermedia*, and *E. allioides*, *E. polycarpa*, respectively.

**Figure 2:**
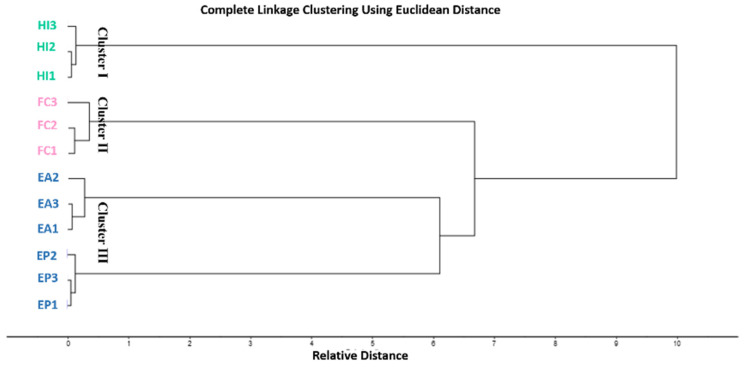
HCA of GC analysis of essential oils of different Apiaceae species based on the identification of the compounds shown in Table 1. *E. allioides* (EA), *E. polycarpa* (EP), *F. clemaditifolia* (FC), and *H. intermedia* (HI).

HCA clustering was performed to have a better insight into species classification. Figure 2 shows the HCA dendrogram for GC fingerprints. HCA dendrogram clustered Apiaceae species into three main clusters. Cluster I, II, and III displayed *F. clemaditifolia*, *H. intermedia* and *E. allioides*, *E. polycarpa*, respectively. The dendrogram confirmed the results obtained from PCA, revealing the similarity of *E. allioides* and *E. polycarpa* as both grouped in one cluster.

### 2.3. Biological Activities

#### 2.3.1. Antibacterial Activity

The disc diffusion and micro-dilution techniques were applied to investigate the antimicrobial activity of the essential oils obtained from the aerial parts of *F. clemaditifolia*, *E. allioides*, *E. polycarpa*, and *H. intermedia*. *Staphylococcus aureus* (ATCC 25923), and *Pseudomonas aeruginosa* (ATCC 85327) were used as representatives of Gram-positive and Gram-negative bacteria, respectively. The mean diameter of inhibition zones (DIZ) and the minimum inhibition concentrations (MIC) values were determined, and the results were presented in Table 2. Three of the studied essential oils showed significantly low antimicrobial activity against the examined bacterial strains with MIC values >500 μg/mL. However, *F. clemaditifolia* oil exerted promising activity comparable to other tested oils with MICs values of 125 µg/mL against the Gram-positive *S. aureus* strains. These results were following previously reported data for the antiinfective activity of the essential oils obtained from six other *Ferula* species that exhibited moderate antimicrobial activity [18,25,26,27]. At the same time, nothing could be found in the literature related to the antibacterial activity of the other two genera *Elaeosticta* or *Hyalolaena*, and here we report for the first time about their biological activities. Although many studies deal with the exact mechanism of antibacterial activities of many essential oils and their components, it is not easy to correlate the activity to a single component. These oils are a complex mixture composed of many active ingredients that work synergistically at many targets in the microbes. Most of the volatile secondary metabolites can diffuse through the bacteria’s cell membranes, leading to destructing many organelles, morphological changes, and interference of the respiratory chain, causing bacterial death [28]. This promising activity of *Ferula* compared to other oils may be attributed to the high content of the oxygenated monoterpenes, especially linalool, citronellol, allo-ocimene, and 4-terpineol. These compounds were reported to have a relevant bactericidal activity against a wide range of Gram-positive and Gram-negative bacteria [29,30,31,32,33].

#### 2.3.2. Antioxidant Activity

The DPPH, ABTS, and FRAP assays were used to determine the in vitro antioxidant activity of the essential oils. The IC50 values of the antioxidant activity of the essential oils obtained from the aerial parts of *F. clemaditifolia*, *E. allioides*, *E. polycarpa*, and *H. intermedia* and ascorbic acid as a positive control were displayed in Table 3. In all the experiments used, *F. clematidifolia* showed superior antioxidant activity with IC50 values of 19.8 and 23.0 μg/mL for the DPPH and ABTS assays, respectively. In the FRAP assay, it exhibited a substantial reducing power of 1308.1 ± 8.9 mM Fe(II)/g. In general, measuring the antioxidant activity by in vitro experiments depends on the power of the sample to scavenge the generated free radicals produced by the system. Phenolic compounds such as acids and flavonoids are the main target secondary metabolites that can strongly suppress these active species. However, oxygenated hydrocarbons can also work in the same context, but to a lesser extent [34]. Regarding the studied samples, the absence of high content of phenolic compounds is the main cause of the moderate antioxidant activity. However, the antioxidant effect may be attributed to the whole oxygenated components of the oils. Similar results for *Ferula* species were explained in previous work based on the mechanism of preventing the free-radical initiation and decomposition of the peroxide radicals [35,36]. Our results are following the previously published data about the antioxidant activity of *Ferula assafoetida* oleo-gum-resin. Both *Ferula* essential oils showed promising antioxidant activity with IC50 values ranging from 10–50 μg/mL [37].

#### 2.3.3. Cytotoxic Activity

The 3-(4,5-dimethylthiazol-2-yl)-2,5-diphenyltetrazolium bromide (MTT) assay was used to assess the cytotoxic activity of the essential oils obtained from *E. allioides*, *E. polycarpa*, *F. clemaditifolia*, *H. intermedia*, and the standard drug doxorubicin against two different cancer cells (HeLa and HepG-2). Most of the evaluated oils showed moderate activity against HeLa cells with IC50 values 138.5–171.2 μg/mL. *F. clemaditifolia* was the most potent cytotoxic agent among these oils against both HeLa and HepG-2 with IC50 values of 138.5 and 252.2 μg/mL, respectively. However, *E. allioides* showed the weakest activity with IC50 values of 233.1 and 387.4 μg/mL, respectively. Nevertheless, it is important to highlight that all the tested oils showed very weak anti-proliferative effect against HepG-2 cells, with IC50 values 252.2–387.4 μg/mL compared to HeLa cells as reported in Table 4. The cytotoxic properties of *F. clemaditifolia* may result from the synergistic action of the different constituents, including limonene, eucalyptol, linalool, 4-terpineol, and citronellol [38,39,40,41,42,43]. These components have been reported to specifically target many vital pathways and processes inside cancer cells, including apoptosis, cell cycle arrest, membrane damage, DNA synthesis inhibition, and ABC transporter modulation [44]. This pattern of antiproliferative activity is typically noticed with *Zanthoxylum* essential oil. This oil has a similar metabolic profile, and the activity was referred to the ability to induce S phase arrest apoptosis and increase the expression of cleaved caspases in HaCaT cells [45].

### 2.4. Partial Least Square Regression Analysis

Partial least square regression (PLS-R) analysis was conducted to establish a link between the gas chromatography fingerprints and their antioxidant activities of various studied species. PLS-R model was constricted by the data matrix X containing the peak area of the GC fingerprints and the response vector y containing the antioxidant activity data. The ideal number of latent variables (LVs) in the PLS model was calculated using minimum root mean square error (RMSE) values obtained by cross-validation (CV). The performance of the model was assessed by the parameters of root mean square error of calibration (RMSEC), root mean square error of validation (RMSEV), and correlation (R^2^). The PLS-R model parameters, including slope, offset, RMSEC, RMSEV, and R^2^, have been shown in Table 5, indicating a superior prediction ability of the PLS regression model. PLS-R models showed excellent linearity and accuracy with R^2^ > 0.99, slope close to 1, intercept close to 0, low RMSEC and RMSEV (close to 0), and low differences between RMSEC and root mean square error of prediction (RMSEP) reveal the robustness of the model. In general, while employing ABTS data, the RMSEV value was less, implying that ABTS findings are more representative than other techniques used to assess antioxidant activity. The prediction performance for the developed models was shown in Table 6. The results showed that the antioxidant activity is correctly predicted with ±5% accuracy.

## 3. Materials and Methods

### 3.1. Plant Material

*Elaeosticta allioides* (Sample No. 27052019340) and *Elaeosticta polycarpa* (Sample No. 27052019335) aerial parts were collected in May 2019 from the Surkhandarya area (Babatag, 38.044681 N; 68.140031 E). *Hyalolaena intermedia* (Sample No. 310520156) was collected in spring 2019 from the Namangan region (Kosonsoy, 41.263346 N; 71.489287 E), while *Ferula clematidifolia* (Sample No. 15062019340) was collected in summer 2019 also from the Surkhandarya area (Babatag, 38.055475 N; 68.154019E), Uzbekistan. Pimenov, M.G. and Malsev I.I. authenticated the plant samples and placed voucher specimens at Uzbekistan National Herbarium.

### 3.2. Essential Oil Isolation

At room temperature, the aerial parts of *E. allioides*, *E. polycarpa*, *H. intermedia*, and *F. clematidifolia* were air-dried in the shade for one week (moisture content not exceeding 10–12%). The dried plant samples (300 g each) were hydro-distilled for 2 h using a Clevenger-type apparatus, yielding 0.14, 0.12, 0.19 and 0.38% *v*/*w*, respectively, after being trapped in dichloromethane. The oils were dried by passing over anhydrous sodium sulfate and kept at −4 °C until being used for further experiments.

### 3.3. Gas Chromatography Analysis

Gas chromatography (GC) was performed on an Agilent 7890B gas chromatograph equipped with a DB-5Ms fused silica column (30 m × 0.25 mm, film thickness 0.25 μm, Agilent Technologies, Middelburg, The Netherlands), which interfaced with an Agilent mass selective detector 5977A (Agilent Technologies, Middelburg, The Netherlands). The interface temperature was 280 °C; the source temperature was 230 °C; ionization energy: 70 eV; and the scan range: 45–950 atomic mass units. A GC autosampler was used to inject 0.5 μL of the sample. The temperature programming was set to supply an oven temperature at 50 °C for 5 min, rising from 50 °C to 280 °C at 5 °C/min, and then finally held isothermally at 280 °C for 15 min. The injector temperature was 250 °C; detector temperature 270 °C; carrier gas helium (0.9 mL/min); and split mode (split ratio, 1:20). The retention indices were calculated based on the retention times of the standard alkane series (C7-C40) purchased from Sigma-Aldrich (Sigma Aldrich GmbH, Sternheim, Germany). Enhanced ChemStation software, version MSD F.01.01.2317 (Agilent Technologies, Middelburg, The Netherlands), was used for recording and integrating the chromatograms. Quantitation was carried out based on the normalization method using the reading of three chromatographic runs. The compounds were identified by comparison of their mass spectral data and their retention indices (RIs) with those reported in the Wiley Registry of Mass Spectral Data (9th Ed.), NIST Mass Spectral Library (2011), and references [46,47].

### 3.4. Biological Evaluation

#### 3.4.1. Antibacterial Assay

The antimicrobial activity was determined using two commonly known standard bacterial strains: *Staphylococcus aureus* (ATCC 25923) as an example of Gram-positive bacteria and *Pseudomonas aeruginosa* (ATCC 85327) as representative of Gram-negative microbes. The antibacterial activity of the essential oils was evaluated using modified agar-disc diffusion and broth micro-dilution techniques (MIC) that were reported previously in details. Briefly, the suspensions of the utilized microorganisms were prepared to a final concentration of approximately 1 × 10^6^ CFU/mL, that followed by inoculating the Mueller Hinton agar (Biomerieux, Marcy l’Étoile, France) with the pathogens. Wells with a diameter of 6 mm were obtained and loaded with 100 μL of 30 mg/mL of essential oil. DMSO, ampicillin (10 μg/mL), and gentamycin (10 μg/mL) were used as controls. The diameters of the growth inhibition zones were determined in triplicate after incubation at 37 °C for 24 h. The MICs of the essential oils were determined by micro-dilution method where the oils were dissolved in 5% DMSO and then were diluted with the broth in 96-well plates to obtain (4–0.007 mg/mL) concentrations. The adjusted bacterial suspension concentrations were then added and the plates were incubated at 37 °C for 24 h (bacteria). The concentration in the first well showed no visible turbidity matching, with a negative control defined as the MIC. Each test was performed in triplicate [48].

#### 3.4.2. Antioxidant Assay

The 2,2-diphenyl-1-picryl-hydrazyl-hydrate (DPPH), 2,2-azino-bis(3-ethylbenzothiazoline-6-sulphonic acid) (ABTS) radical scavenging, and ferric reducing power (FRAP) assays were adopted to determine the in vitro antioxidant activity of the essential oils. The methods were described with all details previously by the same team [49]. In the DPPH assay, the methanol solutions (1 mL) containing 1–500 μg/mL of the essential oils was mixed with 4 mL of a 0.004% methanol solution of DPPH, and the absorbance was measured at 517 nm after incubation in the dark for 30 min at room temperature, while in ABTS assay the diluted methanol essential oils solutions (1 mL) were mixed with freshly prepared ABTS solution (2 mL). The sample absorbance was read at 734 nm after a 30 min incubation at room temperature. However, in FRAP assay, sample solution (0.1 mL) was added to premixed FRAP reagent (2 mL) containing acetate buffer, 2,4,6-tris(2-pyridyl)-s-triazine in HCl and ferricchloride in a ratio of 10:1:1 (*v*/*v*/*v*). Then, the absorbance was detected at 593 nm after a 30 min incubation at room temperature. Citric acid was used as a positive control [50].

#### 3.4.3. Cytotoxicity Assay

The 3-(4,5-dimethylthiazol-2-yl)-2,5-diphenyltetrazolium bromide (MTT) assay was used to assess the cytotoxic activity of the essential oils obtained from *E. allioides*, *E. polycarpa*, *F. clemaditifolia*, *H. intermedia*, and the standard drug doxorubicin against two different cancer cells; HeLa and HepG-2. All the details are mentioned in previous work by the team. The cells (2 × 10^4^ cells per well) were seeded in 96-well plates, and incubated for 24 h. The stock solution (100 mg/mL) of essential oils in DMSO was diluted with the media, and each sample was incubated with cells for 24 h. The MTT solution (0.5 mg/mL) was added and incubated for 4 h. The DMSO (100 μL) was used to dissolve the formazan crystals. The absorbance was measured at 570 nm. Experiments were performed in triplicate [40,51,52,53].

### 3.5. Statistical Analysis

Unless otherwise specified in the technique, all tests were repeated three times. Continuous variables were expressed as mean ± SD. The IC50 value was defined as the concentration of the substance that resulted in a 50% decrease or inhibition of biological activity. A one-way analysis of variance (ANOVA) was used to determine statistical significance, followed by a Tukey’s post hoc test, with a significance level of *p* < 0.05.

### 3.6. Multivariate Analysis

GC-MS data were subjected to multivariate analysis. Principal component analysis (PCA) is the first step in studying the data to offer an overview of all observations and samples to detect and analyse groups, trends, and significant outliers. Hierarchical cluster analysis (HCA) was then employed to enable sample clustering. The clustering patterns were built adopting the entire linkage approach used for group formation; this presentation is more efficient when the Euclidean approach calculates the distance between samples (points). A quantitative calibration model, partial least squares (PLS), was designed to develop a linear relationship between the GC-MS peak area (X) matrix and antioxidant activity (Y) matrix. In this situation, there was no partition of data into model and test set as just 12 samples were assessed (small data set). Therefore, predicting antioxidant activity for fresh samples was not our main concern. The root mean square error (RMSE) and correlation coefficient assessed the PLS-R model capability (R2). PCA, HCA, and PLS were accomplished using CAMO’s Unscrambler^®^ X 10.4 software (Computer-Aided Modeling, As, Norway).

## 4. Conclusions

In this study, the chemical profiles, antimicrobial, cytotoxic, and antioxidant activities of four essential oils obtained from the aerial parts of *Elaeosticta allioides, Elaeosticta polycarpa*, *Hyalolaena intermedia*, and *Ferula clematidifolia* were reported for the first time. Altogether, 100 components were identified in their volatile oils, in which the oxygenated monoterpenes and sesquiterpenes represent the major constituents. Multivariate analysis was adopted to correlate the studied species based on the chemical and biological data, revealing a close relation between *Ferula* and *Elaeosticta*. The partial least square regression model showed excellent prediction results for the antioxidant activity based on GC-MS metabolic profiling. In addition, *Ferula* showed superior biological activity in all experiments that might present a good source for many drug leads, especially for those diseases associated with the high level of oxidative stress. These products are widely accepted among the populations since most of these plants have been used for ages in culinary purposes as food. Therefore, it will be of great value if these plants are applied for therapeutic uses. However, more studies are required to ensure the efficacy and the safety of the drug before its approval.

## Figures and Tables

**Table 1:** Chemical composition of essential oils in the aerial parts of *E. allioides*, *E. polycarpa*, *F. clemaditifolia*, and *H. intermedia*.

Heading	Compound	RI, Calculated	RI, Reported	Peak Area (%)				Method of Identification
				*E. allioides*	*E. polycarpa*	*F. clemaditifolia*	*H. intermedia*	
1	1-Methyl-1,3-cyclohexadiene	771	770	0.19	3.09	tr.	0.47	MS, RI
2	3-Methylenecyclohexene	792	791	0.52	9.46	0.43	1.53	MS, RI
3	2-Hexanol	807	801	-	1.52	-	1.12	MS, RI
4	n-Hexanal	810	810	0.39	5.22	0.62	0.87	MS, RI
5	3-Furaldehyde	834	832	-	-	-	0.42	MS, RI
6	3(Z)-Hexen-1-ol	851	851	0.08	-	0.59	2.14	MS, RI
7	(E)-Salvene	860	865	0.07	2.22	0.14	tr.	MS, RI
8	Santene	886	884	0.08	-	0.01	-	MS, RI
9	Heptanal	900	900	0.26	4.36	-	36.89	MS, RI
10	α-Thujene	929	929	-	-	3.75	-	MS, RI, AU
11	α-Pinene	939	939	0.32	-	1.24	-	MS, RI, AU
12	β-Citronellene	947	947	0.15	-	-	-	MS, RI, AU
13	2(Z)-Heptenal	952	952	-	-	-	1.70	MS, RI
14	Camphene	959	959	0.76	-	-	-	MS, RI, AU
15	1-Heptanol	962	966	-	-	-	1.22	MS, RI
16	Sabinene	966	969	0.23	-	-	tr.	MS, RI, AU
17	Isopropyl tiglate	979	981	0.13	-	0.17	tr.	MS, RI
18	1-Octen-3-one	986	986	-	-	-	2.12	MS, RI
19	β-Pinene	988	988	0.80	-	2.24		MS, RI, AU
20	2,3-Dehydro-1,8-cineole	997	994	-	-	0.08		MS, RI
21	Octanal	1002	1002	1.80	-	-	1.71	MS, RI
22	2,4-(E,E)-Heptadienal	1011	1011	-	-	-	0.37	MS, RI
23	α-Phellandrene	1013	1013	-	tr.	0.09	tr.	MS, RI, AU
24	1-(1-Cyclohexen-1-yl)-ethanone	1028	1023	-	-	-	1.47	MS, RI
25	p-Cymene	1029	1029	tr.	-	5.09	tr.	MS, RI, AU
26	D-Limonene	1034	1034	2.83	tr.	9.42	0.48	MS, RI, AU
27	Eucalyptol	1039	1039	-	-	-	0.28	MS, RI, AU
28	β-(Z)-Ocimene	1044	1045	0.57	-	0.22	-	MS, RI
29	Benzeneacetaldehyde	1046	1046	-	tr.	-	1.44	MS, RI
30	β-(E)-Ocimene	1052	1052	-	2.14	-	tr.	MS, RI
31	(3Z)-Octen-1-ol	1056	1058	-	tr.	--	4.73	MS, RI
32	γ-Terpinene	1062	1062	0.27	-	4.8	-	MS, RI, AU
33	cis-Linalool oxide	1073	1073	0.28	-	2.19	-	MS, RI
34	trans-Linalool oxide	1090	1090	0.31	-	-	-	MS, RI
35	p-Mentha-1,4(8)-diene	1092	1092	-	1.20	0.88	0.97	MS, RI
36	Linalool	1099	1099	1.35	4.11	-	1.33	MS, RI, AU
37	tetrahydro-Linalool	1103	1099	0.21	2.92	9.02	0.8	MS, RI
38	cis-Thujone	1107	1102	-	-	2.01	-	MS, RI
39	trans-Thujone	1112	1112	-	1.98	-	-	MS, RI
40	allo-Ocimene	1126	1127	0.15	tr.	14.55	0.22	MS, RI
41	p-Menth-2-en-1-ol	1130	1130	0.08	-	0.16	-	MS, RI
42	cis-p-Mentha-2,8-dien-1-ol	1140	1141	0.16	-	-	-	MS, RI
43	(E)-2-Nonenal	1159	1159	0.28	tr.	-	4.24	MS, RI
44	Borneol	1165	1165	0.21	-	0.15	-	MS, RI, AU
45	Menthol	1167	1171	-	-	-	1.71	MS, RI, AU
46	4-Terpineol	1174	1174	0.19	-	6.39	-	MS, RI, AU
47	α-Terpineol	1186	1186	0.28	-	3.33	-	MS, RI, AU
48	trans-Carveol	1223	1223	0.41	-	-	-	MS, RI
49	β-Cyclocitral	1225	1224	-	-	-	0.33	MS, RI
50	Citronellol	1229	1229	-	-	6.94	-	MS, RI
51	Neral	1239	1239	0.04	-	0.07	0.31	MS, RI, AU
52	Geraniol	1249	1250	0.56	-	0.31	0.79	MS, RI, AU
53	Linalool acetate	1253	1254	0.04	-	0.04	0.36	MS, RI
54	2(E)-Decenal	1262	1263	0.83	-	-	1.03	MS, RI
55	dihydro-Linalool acetate	1275	1271	0.06	2.41	0.07	0.50	MS, RI
56	Bornyl acetate	1290	1290	3.60	-	0.06	-	MS, RI
57	Undecanal	1316	1313		1.29	-	1.53	MS, RI
58	p-Mentha-1,4-dien-7-ol	1329	1329	-	6.71	0.12	1.01	MS, RI
59	δ-Terpinyl acetate	1340	1340	0.09	1.57	0.11	0.57	MS, RI
60	α-Longipinene	1355	1355	0.11	-	0.12	-	MS, RI
61	β-Patchoulene	1387	1382	0.31	-	0.24	-	MS, RI
62	β-Bourbonene	1397	1394	0.52	-	-	-	MS, RI
63	β-Caryophyllene	1433	1433	0.54	-	1.4	0.38	MS, RI, AU
64	Aromandendrene	1449	1447	0.08	-	0.06	-	MS, RI, AU
65	α-Humulene	1469	1469	0.25	-	0.26	-	MS, RI
66	γ-Muurolene	1485	1486	1.35	-	0.28	1.17	MS, RI
67	Germacrene D	1494	1496	16.09	-	-	-	MS, RI
68	α-Cuprenene	1503	1505	tr.	-	tr.	-	MS, RI
69	Germacrene A	1506.4	1506	1.51	-	tr.	0.27	MS, RI
70	γ-Cadinene	1509	1509	-	-	1.16	-	MS, RI
71	Cubebol	1513	1513	0.21	-	-	0.52	MS, RI
72	δ-Cadinene	1522	1522	tr.	-	-	1.1	MS, RI
73	γ-Cuprenene	1527	1523	2.12	-	0.61		MS, RI
74	α-Cadinene	1533	1538	0.32	3.41	0.11	tr.	MS, RI
75	(-)-Spathulenol	1577	1576	2.51	-	-		MS, RI
76	Caryophyllene oxide	1582	1582	1.45	-	0.82	0.37	MS, RI, AU
77	Salvial-4(14)-en-1-one	1607	1604	2.46	-	-	-	MS, RI
78	Humulene epoxide II	1621	1620	1.80	-	-	-	MS, RI
79	β-Himachalene oxide	1627	1613	1.59	tr.	-	-	MS, RI
80	1-epi-Cubenol	1638	1632	1.18	-	0.06	-	MS, RI
81	β-Eudesmol	1657	1654	2.51	-	-	-	MS, RI
82	α-Cadinol	1667	1667	2.06	-	0.08	-	MS, RI
83	β-Bisabolol	1676	1675	3.04	-	-	-	MS, RI
84	Apiole	1678	1679	-	-	0.27	0.57	MS, RI
85	α-Bisabolone oxide A	1688	1686	-	-	0.15	-	MS, RI
86	α-Bisabolol	1690	1695	4.17	-	-	0.62	MS, RI, AU
87	Germacrone	1696	1696	-	1.72	0.36	tr.	MS, RI
88	Caryophyllene acetate	1704	1704	4.25	-	-	-	MS, RI
89	Cedr-8(15)-en-9α-ol, acetate	1745	1743	0.37	3.14	0.08	0.64	MS, RI
90	α-Bisabolol oxide A	1753	1746	0.37	1.14	-	-	MS, RI
91	Benzyl benzoate	1779	1775	tr.	2.84	0.06	0.66	MS, RI
92	β-Bisabolenal	1785	1765	4.07	-		-	MS, RI
93	Cedryl methyl ketone	1791	1791	1.00	-	0.03	-	MS, RI
94	β-Bisabolenol	1806	1790	1.41	-	0.08	-	MS, RI
95	Methyl-hexadecanoic acid ester	1923	1924	1.18	-	1.3	-	MS, RI
96	Hexadecanoic acid	1956	1958	0.71	4.83	1.58	2.67	MS, RI
97	Phytol	2110	2112	0.18	2.57	0.33	-	MS, RI
98	Methyl stearate	2125	2128	0.46	1.48	0.95	-	MS, RI
99	Ethyl octadecanoate	2165	2188	0.05	1.98	0.06	-	MS, RI
100	Hinokiol	2590	2590	0.16	7.72	0.04	0.86	MS, RI
	Monoterpenes			5.93	3.34	42.28	1.67	
	Oxygenated monoterpenes			8.02	19.7	31.05	7.99	
	Sesquiterpenes			23.2	3.41	4.24	2.92	
	Oxygenated sesquiterpenes			34.45	6	1.66	2.15	
	Alkenes			0.80	11.68	0.75	1.53	
	Alcohols			0.42	11.81	1.23	10.64	
	Aldehyde and ketones			3.56	10.87	0.62	53.79	
	Fatty acids and their esters			2.40	8.29	3.89	2.67	
	Others				2.84	0.06	0.66	
	Total identified			78.97	81.03	85.78	84.49	

Compounds were identified based on the compounds’ mass spectral data (MS), and retention indices (RI), compared with those of the NIST Mass Spectral Library (December 2011), the Wiley Registry of Mass Spectral Data, 8th edition, and many authentic standards (AU). The content (%) was calculated using the normalization method based on the GC-MS data. The presented data is the average of three replicas (tr. = trace, concentration less than 0.01%)

**Table 2:** Minimum inhibitory concentrations (MIC) in (µg/mL) and mean diameter of inhibition zones (DIZ) in (mm) of *E. allioides*, *E. polycarpa*, *F. clemaditifolia*, and *H. intermedia* essential oils against different pathogens determined by microdilution and agar diffusion method.

Microorganisms	*E. allioides*		*E. polycarpa*		*F. clematidifolia*		*H. intermedia*		Positive Control *	
	DIZ (mm)	MIC (µg/mL)	DIZ (mm)	MIC (µg/mL)	DIZ (mm)	MIC (µg/mL)	DIZ (mm)	MIC (µg/mL)	DIZ (mm)	MIC (µg/mL)
Gram-positive bacteria										
*S. aureus* ATCC 25923	10.8 ± 0.4	>500	11.7 ± 0.5	>500	19.4 ± 0.1	125	11.8 ± 0.6	>500	30.1 ± 0.6	0.16
Gram negative bacteria										
*P. aeruginosa* ATCC 85327	8.3 ± 0.2	>500	9.3 ± 03	>500	9.6 ± 0.4	250	15.1 ± 0.3	>500	24.7 ± 0.3	0.24

Data are presented as means ± S.D. *n* = 3; * The positive control was taken as ampicillin for Gram-positive bacteria and gentamycin for Gram-negative bacteria; In all assays, 30 mg essential oil, 10 µg of the standard antibiotic in 1 mL DMSO, and 100 μL were applied. DIZ, the diameter of inhibition zone is measured in (mm) by the agar diffusion method.

**Table 3:** IC50 values (μg/mL) for the antioxidant activities of the essential oils obtained from *E. allioides*, *E. polycarpa*, *F. clemaditifolia*, and *H. intermedia* using the DPPH, ABTS, and FRAP assays.

Sample	DPPH	ABTS	FRAP
	IC50 (μg/mL)	IC50 (μg/mL)	mM FeSO_4_/g Sample
*E. allioides*	95.1 ± 3.6	102.7 ± 0.9	1034.2 ± 16.4
*E. polycarpa*	136.0 ± 8.9	127.8 ± 6.4	998.8 ± 11.2
*F. clematidifolia*	19.8 ± 0.6	23.0 ± 1.7	1308.1 ± 8.9
*H. intermedia*	35.3 ± 2.9	39.2 ± 1.0	843.5 ± 6.7
Ascorbic acid	0.27 ± 0.0	6.9 ± 0.2	3421.3 ± 29.3

Data are presented as means ± S.D. *n* = 3.

**Table 4:** IC50 values (μg/mL) for the cytotoxic activity of the essential oils obtained from *E. allioides*, *E. polycarpa*, *F. clemaditifolia*, and *H. intermedia* against HeLa and HepG-2 cell lines using the MTT assay.

Sample	HeLa	HepG-2
*E. allioides*	233.1 ± 8.6	387.4 ± 13.0
*E. polycarpa*	156.3 ± 10.2	298.1 ± 9.1
*F. clematidifolia*	138.5 ± 3.9	252.2 ± 12.4
*H. intermedia*	171.2 ± 10.3	291.7 ± 15.9
Doxorubicin (µM)	2.1 ± 0.14	0.78 ± 0.05

Data are presented as means ± S.D. *n* = 3.

**Table 5:** The partial least square regression model parameters used for prediction.

Antioxidant Activity	Data Type	PLS			
		Slope	Offset	RMSE	R^2^
DPPH	Cal.	0.9999	0.0065	0.4452	0.9999
	Val.	0.9995	0.0401	0.6581	0.9998
ABTS	Cal.	0.9999	0.0055	0.3760	0.9999
	Val.	0.9995	0.0415	0.5542	0.9998
FRAP	Cal.	0.9999	0.0307	0.9083	0.9999
	Val.	0.9995	0.3915	1.3430	0.9999

RMSE, root mean squared error; R^2^, Correlation; Cal., Calibration; Val., Validation.

**Table 6:** Results of calibration and predictive ability of the partial least square model.

Heading	DPPH		ABTS		FRAP	
	Y Reference	Y Predicted	Y Reference	Y Predicted	Y Reference	Y Predicted
FC1	19.8	19.71874	23	22.94632	1308.1	1308.702
FC2	19.4	20.47383	22.8	23.69492	1307.9	1305.79
FC3	20.2	19.22362	23.3	22.47157	1309.2	1310.675
EA1	95.1	95.12069	102.7	102.7001	1034.2	1034.095
EA2	94.99	95.0702	102.4	102.6058	1034	1034.468
EA3	95.2	95.09119	102.9	102.6873	1034.5	1034.145
EP1	136	136.0015	127.8	127.7339	998.8	998.6842
EP2	135.8	135.6428	127.4	127.4543	998.5	999.4387
EP3	136.2	136.3489	128	128.0072	998.9	998.0963
HI1	35.3	35.28377	39.2	39.18572	843.5	843.4024
HI2	35	35.32335	39	39.24232	843.2	843.9583
HI3	35.6	35.29141	39.4	39.17062	843.8	843.1453

## Data Availability

All the data are available via the corresponding authors upon request.
